# The Key Genes for Perineural Invasion in Pancreatic Ductal Adenocarcinoma Identified With Monte-Carlo Feature Selection Method

**DOI:** 10.3389/fgene.2020.554502

**Published:** 2020-10-15

**Authors:** Jin-Hui Zhu, Qiu-Liang Yan, Jian-Wei Wang, Yan Chen, Qing-Huang Ye, Zhi-Jiang Wang, Tao Huang

**Affiliations:** ^1^Department of General Surgery, The Second Affiliated Hospital, Zhejiang University School of Medicine, Hangzhou, China; ^2^Department of General Surgery, Jinhua People’s Hospital, Jinhua, China; ^3^Department of Surgical Oncology, The Second Affiliated Hospital, Zhejiang University School of Medicine, Hangzhou, China; ^4^Shanghai Institute of Nutrition and Health, Shanghai Institutes for Biological Sciences, Chinese Academy of Sciences, Shanghai, China

**Keywords:** perineural invasion, pancreatic ductal adenocarcinoma, Monte-Carlo feature selection, incremental feature selection, support vector machine

## Abstract

**Background:**

Pancreatic ductal adenocarcinoma (PDAC) is the most aggressive form of pancreatic cancer. Its 5-year survival rate is only 3–5%. Perineural invasion (PNI) is a process of cancer cells invading the surrounding nerves and perineural spaces. It is considered to be associated with the poor prognosis of PDAC. About 90% of pancreatic cancer patients have PNI. The high incidence of PNI in pancreatic cancer limits radical resection and promotes local recurrence, which negatively affects life quality and survival time of the patients with pancreatic cancer.

**Objectives:**

To investigate the mechanism of PNI in pancreatic cancer, we analyzed the gene expression profiles of tumors and adjacent tissues from 50 PDAC patients which included 28 patients with perineural invasion and 22 patients without perineural invasion.

**Method:**

Using Monte-Carlo feature selection and Incremental Feature Selection (IFS) method, we identified 26 key features within which 15 features were from tumor tissues and 11 features were from adjacent tissues.

**Results:**

Our results suggested that not only the tumor tissue, but also the adjacent tissue, was informative for perineural invasion prediction. The SVM classifier based on these 26 key features can predict perineural invasion accurately, with a high accuracy of 0.94 evaluated with leave-one-out cross validation (LOOCV).

**Conclusion:**

The in-depth biological analysis of key feature genes, such as TNFRSF14, XPO1, and ATF3, shed light on the understanding of perineural invasion in pancreatic ductal adenocarcinoma.

## Introduction

Pancreatic cancer is a type of common malignant tumor of the digestive tract, the most aggressive form of which is pancreatic ductal adenocarcinoma (PDAC), which has a 5-year survival rate of only 3–5% (Huang et al., 2014). The poor prognosis of PDAC is largely due to the lack of early symptoms, explosive outcomes, and resistance to treatment (Pour et al., 2003).Currently, there is no effective method to detect pancreatic cancer in its early stages. However, with the increasing insight into the mechanism of this cancer over time, novel therapies are being researched and developed (Rossi et al., 2014).

Pancreatic cancer has poor responses to conventional therapies, such as chemotherapy and irradiation (Rossi et al., 2014). Although surgery has been indicated to be an effective therapeutic approach to eliminate cancer cells, 70–81% of patients are rendered unresectable because of locally advanced disease or distant metastatic lesions (White et al., 2001; Mossner, 2010; Cai et al., 2013) and most patients who have undergone surgery experience recurrence and comorbidities (Pour et al., 2003). In the last few decades, Gemcitabine has been the preferred treatment option for PDAC. However, recent studies suggested that FOLFIRINOX (a regimen combining fluorouracil, leucovorin, oxaliplatin, and irinotecan) has shown a significant therapeutic advantage in patients with advanced PDAC (Kleger et al., 2014; Ferrone et al., 2015). In addition, the curative effect of oral fluorouracil in Asian patients with PDAC has been proven (Cid-Arregui and Juarez, 2015).

Most studies have focused on biomarkers to predict the progression or recurrence of PDAC. It has been reported that about 90% of the later stage pancreatic cancers have point mutations of KRAS, indicating that KRAS may be used as a diagnostic marker of PDAC (Campbell et al., 2007; De Oliveira et al., 2012; Zhang et al., 2014). SLIT2-ROBO signaling in PDAC has also been reported to enhance metastasis and predispose PDAC cells to neural invasion (Gohrig et al., 2014). There have also been some important and highly penetrative genes identified, such as CEACAM1, MCU, VDAC1, PKM2, CYCS, C15ORF52, TMEM51, LARP1, and ERLIN2 (Calabretta et al., 2016; Giulietti et al., 2016). Although many biomarkers have now been shown to be associated with PDAC, their effectiveness in the early detection of cancer still require verification.

Perineural invasion (PNI) is a process in which cancer cells invade the surrounding nerves and perineural spaces (Ceyhan et al., 2008), which is associated with recurrence (Dai et al., 2007; Gil et al., 2010) and poor outcome (Bapat et al., 2011). PNI also contributes to the severe pain syndrome in patients with advanced PDAC (Zhu et al., 1999; Esposito et al., 2008). It is estimated that the incidence of PNI reaches up to 90% in pancreatic cancer (Nakao et al., 1996). The high incidence of PNI in pancreatic cancer limits radical resection and promotes local recurrence, which negatively affects life quality and survival time of the patients with pancreatic cancer (Hirai et al., 2002). Among the factors influencing the prognosis of pancreatic cancer, PNI has gradually become an independent prognostic factor and pathological feature. Therefore, further studies are urgently needed to investigate the mechanism of PNI in pancreatic cancer, thus providing a theoretical basis for the treatment of pancreatic cancer. Adjacent tissues are important parts of a tumor microenvironment, and Existing studies have taken adjacent tissues as normal tissues for control to study the difference between cancer tissues and normal tissues. However, present studies have indicated that there will still be some physiological changes in adjacent tissues affected by the tumor tissues of patients (Casbas-Hernandez et al., 2015; Yamakawa et al., 2019). A number of studies have included adjacent tissues in cancer research, and researchers have found that adjacent tissues can also serve as a marker of tumor prognosis (Lee et al., 2019). In this study, PNI was studied in combination with the differences between tumor tissues and adjacent tissues of patients to find prognostic biomarkers affecting PNI.

In this work, we analyzed the gene expression profiles of 28 pancreatic ductal adenocarcinoma patients with perineural invasion and 22 pancreatic ductal adenocarcinoma patients without perineural invasion. Both tumor and adjacent tissues were profiled. With Monte-Carlo feature selection and Incremental Feature Selection (IFS) method, 26 key features were identified. Interestingly, 15 of them were from tumor tissues but the other 11 features were from adjacent tissues. Our results proved that the microenvironment of the tumor is important for perineural invasion. Based on these 26 key features, a Support Vector Machine (SVM) predictor was constructed and its accuracy, evaluated with Leave-One-Out Cross Validation (LOOCV), was 0.94, which needs to be validated in another independent large dataset. But many key feature genes, such as TNFRSF14, XPO1, and ATF3, showed great promise on explaining perineural invasion in pancreatic ductal adenocarcinoma.

## Materials and Methods

### Datasets

We downloaded the gene expression profiles of tumors and adjacent tissues in 50 pancreatic ductal adenocarcinoma patients from GEO (Gene Expression Omnibus) under accession number GSE102238 (Yang et al., 2020). In this dataset, 28 patients had perineural invasion while 22 patients had an absence of perineural invasion. Each patient had both tumor and adjacent samples. The gene expression levels were profiled with 25,492 probes from the Agilent-052909 CBC_lncRNAmRNA_V3 platform, which included both lncRNAs and mRNAs.

To systematically compare the difference between pancreatic ductal adenocarcinoma patients with perineural invasion and pancreatic ductal adenocarcinoma patients without perineural invasion, we combined the gene expression levels from tumor samples and adjacent samples for each patient. Therefore, there were 25,492 × 2 = 50,984 gene expression features. Our goal was to identify the key genes from either tumor or adjacent samples that could discriminate the patients with perineural invasion and without perineural invasion.

### Identification of Key Genes Using Monte-Carlo Feature Selection

As we can see, the feature number was much greater than the sample size. If we directly used so many features to classify the patients, it would obviously overfit. To partially solve this problem, we adopted the Monte-Carlo feature selection method (Draminski et al., 2008) to rank the features. The Monte-Carlo feature selection method randomly chooses a number of features and then constructs a number of tree classifiers (Chen et al., 2018a; Pan et al., 2018; Wang et al., 2018). Based on these tree classifiers, it assigns each feature an importance value. If a feature is selected by many tree classifiers, it is more important than others.

Let us formulate the algorithm formally. Suppose d is the total number of features, we randomly select m features (m≪d) for s times and construct t trees for each of the s subsets. At last, s⋅t classification trees will be constructed. A feature g’s relative importance (RI) can be reflected by how many times it is used to set a decision rule by the s⋅t trees and how much it contributes to the classification of the s⋅t trees, and is calculated with the equation below:

(1) $$R\,I_{g}=\sum_{\tau =1}^{\mathrm{st}}{\left(\mathrm{wAcc}\right)}^{u}\,\sum_{n_{g}\,\left(\tau \right)}I\,G\,\left(n_{g}\,\left(\tau \right)\right)\,{\left(\frac{n\,o.i\,n\,n_{g}\,\left(\tau \right)}{n\,o.i\,n\,\tau }\right)}^{v}$$

where *wAcc* is the weighted classification accuracy of decision treeτ, *IG*(*n*_*g*_(τ) is the information gain of node *n*_g_(τ), *no*.*inn*_*g*_(τ) is the number of samples under node *n*_g_(τ), *no*.*in*τ is the number of samples in decision tree and τ, u and v are parameters.

To be more specific, wAcc is defined as follows:

(2) $$\mathrm{wAcc}=\frac{1}{c}\,\sum_{i=1}^{c}\frac{n_{i\,i}}{n_{i\,1}+n_{i\,2}+\cdots +n_{i\,c}}$$

where c is the number of classes (it is 2 in this study) and *n*_ij_ is the number of samples from class i that are classified as class j(i,j=1,2,…,c)

The features were ranked based on their RI values from large to small as F

(3) $$F=\left[f_{1},f_{2},\ldots ,f_{N}\right]$$

where *N* is the total number of features (50,984 for this study).

### Construction of SVM Predictor for Perineural Invasion

Although all features were ranked using Monte-Carlo feature selection, it was not clear how many top features should be selected to construct a final predictor for perineural invasion. To choose the final key features for the predictor, we adopted the Incremental Feature Selection (IFS) method (Wang et al., 2017; Zhang et al., 2017; Chen et al., 2018b,c; Li et al., 2018) to optimize the key features and their predictor. We tested 500 different feature sets (*F*_1_,*F*_2_,…,*F*_500_), where*F*_*i*_ = [*f*_1_,*f*_2_, …,*f*_*i*_]. In other words, feature set *F*_*i*_ contains the top i features in F from equation (2). For each feature set, we constructed a support vector machine (SVM) predictor. Based on the number of features and their accuracy, we can balance the model complexity and performance and choose the final key features and optimized model. In this study, the SVM predictor was constructed using R function svm from package e1017 and leave-one-out cross validation (LOOCV) was used to evaluate the performance of the SVM predictor.

## Results and Discussion

### The Top Discriminative Genes Between Patients Were With Perineural Invasion and Without Perineural Invasion

The gene expression profiles in the tumor and adjacent tissues can represent the difference between pancreatic ductal adenocarcinoma patients with perineural invasion and without perineural invasion. The gene expression in the tumor directly shows the activity of pancreatic ductal adenocarcinoma while the gene expression in the adjacent tissue reflect the microenvironment of the tumor. Therefore, we combined the gene expression profiles in tumors and in adjacent tissues for each patient and compared the combined profiles between pancreatic ductal adenocarcinoma patients with perineural invasion and without perineural invasion using Monte-Carlo feature selection. Based on the RI values, which represented how well a gene feature can classify the two groups of patients, we ranked all the features and further analyzed the top 500 discriminative genes.

### The Final Key Features and SVM Predictor for Perineural Invasion

**FIGURE 1:**
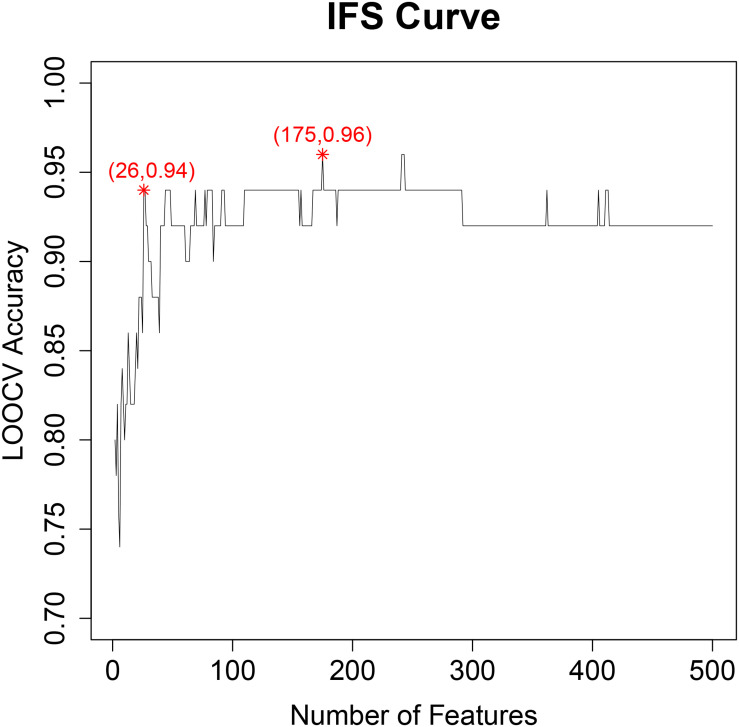
The IFS curve for final key feature selection. The *X*-axis was the number of features. The *Y*-axis was their prediction accuracy evaluated with LOOCV. When 175 genes were used, the accuracy was the highest, at 0.96. But when only 26 genes were used, the accuracy became 0.94. Balancing both model complexity and performance, we chose the 26 genes as the final key features and their SVM predictor as the optimized predictor for perineural invasion.

With IFS method (Chen et al., 2017a,b,c,d; Li and Huang, 2017; Liu et al., 2017), we evaluated the prediction accuracy of different feature sets and plotted the IFS curve in which the *X*-axis was the number of features and the *Y*-axis was their prediction accuracy evaluated with LOOCV. The IFS curve was shown in Figure 1. It can be seen that when 175 genes were used, the accuracy was the highest, at 0.96. But when only 26 genes were used, the accuracy became 0.94. Balancing both model complexity and performance, we chose the 26 genes as the final key features and their SVM predictor as the optimized predictor for perineural invasion. The 26 key features were given in Table 1. With the 26 key features, 15 features were from tumor tissues while 11 features were from adjacent tissues. These results suggested that not only the tumor tissue, but also the adjacent tissue, was informative for perineural invasion prediction.

**TABLE 1:** The 26 key discriminative features between patients with perineural invasion and without perineural invasion.

Rank	Tissue	Probe	RI	Probe sequence	blastn Score	blastn Identities	Chromosome position	Gene symbol
1	Adjacent	p12601	0.0518	GGCTGAAGGAGAACTTCAATATCATATATTTTAAA GGTTGACTCACAGTTTGGAACAAGA	111bits (60)	60/60 (100%)	chr4:3545147-3545206	AL590235.1
2	Tumor	p18602	0.0395	CTTGCCATTTAGCCACTGTTACTGATAATTTGGAT GGAAAAGAAAAATAAATTATATCAC	111bits (60)	60/60 (100%)	chr12:25944921-25944862	RASSF8-AS1
3	Tumor	A_24_P88801	0.0370	CAGACGGAGTTCATGAACCTTTTGACCTTTCAG AGCAGACCTATGACTTCTTGGGTGAAA	111bits (60)	60/60 (100%)	chr2:110123870-110123811	NPHP1
4	Tumor	A_33_P3329128	0.0352	GGTGGCTGGACAATCATTTGCTAAAGATATT AGGCATGTGACTCATGAATCCAATCAATC	No significant similarity found			
5	Adjacent	A_21_P0010506	0.0271	TTAGGCCAAGTGTGGAGAAATCAATGATGT TGACGATGAGGCTCCCTGAGAGAAATCACA	111bits (60)	60/60 (100%)	chr1:2565049-2565108	TNFRSF14
6	Adjacent	A_24_P941759	0.0243	ATGCTTCAAGTAATGCAATACAAAACATA ACCCATTTAATGATGAATTACTTGAATGTAT	111bits (60)	60/60 (100%)	chr14:30619533-30619592	G2E3
7	Adjacent	p14843	0.0238	AGTGACTGATTTGAAACCAGTTGTACCGTA TTATTAGGAAAGGCGCCTTGATGAAAAGAT	80.5 bits (43)	43/43 (100%)	chr7:19813727-19813685	AC004543.1
8	Adjacent	p28694	0.0238	CATTTACTGGGCCTGAAATGGGAAAATGAA AGATGTGGCAAGAAACTGACAAGGGCCCAA	111bits (60)	60/60 (100%)	chr1:1660100-1660159	FO704657.1/SLC35E2B
9	Tumor	p4684	0.0236	AAGGTGTTGAAGCATAGACGCTGGAACATAAAATG ACTCATGATCTCACTGGGAGAAGGG	111bits (60)	60/60 (100%)	chr14:101635450-101635391	LINC02320
10	Adjacent	A_23_P170088	0.0214	ATCCTTTCTGTGCTGCTTTAGGCATCTGCC CTTACGTGGTTCGTGTCCAGCTCTGTCAAC	106 bits (57)	59/60 (98%)	chr9:137392642-137392583	EXD3
11	Tumor	A_23_P398372	0.0198	TCAGGTAGAGAAAGCAAAAAATCTCTGGCCGTAA ACCGTGCTCTCTAATTTATCGGCAGC	111bits (60)	60/60 (100%)	chr9:136115009-136114950	TMEM250
12	Tumor	A_24_P277673	0.0191	CAAGGTACTGAGCGATAATATTCAGGGCATTACC AAGTGCACTATCCGGCGCTTGGCCCG	111bits (60)	60/60 (100%)	chr6:26246918-26246859	HIST1H4G
13	Tumor	A_19_P00803575	0.0190	AAGCAGCTTGTATAATTCCAACTGGTGTTT CATTTCTGTTCTAATGCTAAGTGGTAACGC	111bits (60)	60/60 (100%)	chr17:17192194-17192253	MPRIP
14	Tumor	A_33_P3802966	0.0190	GAGGGGGTTAACATAACGCGGACCGATCCCA AATGGCATTGATGAGTGTACCTCCCACGA	No significant similarity found			
15	Adjacent	A_32_P207124	0.0166	CCCTGCCCTTCCCATCTTAGGGTGTCGTCT GAGACAGACTCTTATTCCCTCAATAAAGAG	111bits (60)	60/60 (100%)	chrX:120932228-120932169	CT47A12
16	Tumor	A_23_P410128	0.0165	AAACCAACAAATAAAAGCATGATAAATTGACT ATATCAAAATTTAAAACTTCTCTATGAC	111bits (60)	60/60 (100%)	chr22:42027707-42027766	WBP2NL
17	Adjacent	p28485	0.0157	AGTCTCAGGGCTAGACGTATTCCAAATATTTGGAT AATTCAAAGTAATTTGCACAGACAT	111bits (60)	60/60 (100%)	chr7:28738207-28738148	CREB5
18	Tumor	p13289	0.0155	CTAGGGTGCTCTATGCTGTGATGCTATCAAATC TTCATGGATTTTTCCAGGATCCTCAAA	111bits (60)	60/60 (100%)	chr5:92432262-92432203	AC114316.1/AC124854.1
19	Tumor	A_33_P3349334	0.0149	CAGGCTTGTATGATCTATTCCTTACCACAAAAG AAGTAGACAATTGCCACTTTTATTTCT	111bits (60)	60/60 (100%)	chr14:55049328-55049387	SOCS4
20	Adjacent	A_23_P40078	0.0146	GGGTATTTGTCGACCAAAATGATGCCAATTTG TAAATTAAAATGTCACCTAGTGGCCCTT	111bits (60)	60/60 (100%)	chr2:61478760-61478701	AC016727.1/XPO1
21	Adjacent	RNA143544| tRNA_461_68	0.0145	AGAAAAACCATTTCATAACTTTGTCAAAGTTAAA TTATAGGCTAAATCCTATATACCTTA	106 bits (57)	59/60 (98%)	chrM:7526-7585	MT-TD
22	Tumor	A_33_P3259817	0.0142	AGAAATGAAAGCCAACTACAGGGAAATGGC GAAGGAGCTTTCTGAAATCATGCATGAGCA	111bits (60)	60/60 (100%)	chr13:98797175-98797116	DOCK9
23	Tumor	RNA95815| RNS_897_109	0.0141	TAAAAGGAGAAAGGGAGGGGCCTTGTGAGGTG AAGGGTGTCCTTATACAGGTGTGACAGC	111bits (60)	60/60 (100%)	chr20:56660085-56660144	
24	Adjacent	A_24_P33895	0.0139	TCCAGAAGATGAGAGAAACCTCTTTATCCAACA GATAAAAGAAGGAACATTGCAGAGCTA	111bits (60)	60/60 (100%)	chr1:212619495-212619554	ATF3
25	Adjacent	p29186	0.0138	AGGCGGGGGATGCTGTGTGGCACCTCCTATTG TCTCTTTTTGCGTTTTCTCCCATTCTCG	111bits (60)	60/60 (100%)	chr1:19188401-19188460	UBR4
26	Tumor	A_33_P3351785	0.0137	GCATCAAAATCAACAAAAAACCAGAATATAGTC CCAAAAGAGAAATCCACCAAGTACCAT	111bits (60)	60/60 (100%)	chr20:8871665-8871724	PLCB1

### Compare the SVM Predictor With Other Classification Methods

To compare the SVM predictor with other classification methods, we tried three other classification algorithms: decision tree (R function rpart from package rpart), nearest neighbor (R function knn with *k* = 1 from package class), and naïve Bayes (R function naiveBayes from package e1071). The highest accuracies of decision tree, nearest neighbor, naïve Bayes were 0.76 with 24 features, 0.94 with 44 features, and 0.94 with 185 features, respectively. Their performances were worse than SVM and required more features.

### Compare the Monte-Carlo Feature Selection With Other Seven Feature Selection Methods

There have been many feature selection methods. Each has its assumption and application scenario. Therefore, we compared the Monte-Carlo feature selection results with seven other feature selection methods in Weka (Frank et al., 2016): chi-squared statistic (ChiSquared), correlation (Correlation), gain ratio (GainRatio), information gain (InfoGain), OneR classifier (OneR), ReliefF (ReliefF), and symmetrical uncertainty (SymmetricalUncert). The default parameters in Weka were used for the seven feature selection methods.

We checked the ranks of the 26 key features selected by the Monte-Carlo method in the other seven feature selection methods. Their ranks were listed in Table 2. It can be seen that most of the features ranked on top with other methods as well. The first feature by Monte-Carlo ranked fourth by OneR, the third feature ranked second by GainRatio, the fourth feature ranked fourth by GainRatio, the fifth feature ranked fifth by SymmetricalUncert, the sixth feature ranked fourth by ReliefF, the seventh feature ranked eight by Correlation, and the eighth feature ranked third by SymmetricalUncert.

**TABLE 2:** Ranking of the 26 key features selected by the Monte-Carlo method in the other seven feature selection methods.

Monte Carlo	Best Rank in other seven methods	ChiSquared	Correlation	GainRatio	InfoGain	OneR	ReliefF	SymmetricalUncert
1	OneR 4	6	12	51	12	4	21	15
2	ChiSquared 14	14	49	52	24	94	306	30
3	GainRatio 2	18	1121	2	16	267	11	9
4	GainRatio 4	20	501	4	15	227	220	8
5	SymmetricalUncert 5	13	38	13	10	527	1602	5
6	ReliefF 4	26	43	168	39	613	4	67
7	Correlation 8	10	8	135	19	1839	80	29
8	SymmetricalUncert 3	11	74	12	9	6306	121	3
9	ChiSquared 25	25	47	170	38	5466	194	69
10	Correlation 5	40	5	140	48	760	545	72
11	GainRatio 1	19	1205	1	18	10	67	11
12	GainRatio 29	123	1697	29	114	11583	8543	46
13	Correlation 1	31	1	17	13	73	3	14
14	Correlation 6	45	6	9	31	1037	160	22
15	ChiSquared 1, InfoGain 1, SymmetricalUncert 1	1	89	11	1	13	7	1
16	SymmetricalUncert 4	12	239	14	11	4030	128	4
17	ReliefF 38	100	2787	238	155	1528	38	269
18	ChiSquared 53	53	106	417	57	366	572	192
19	GainRatio 3	17	1935	3	17	609	115	10
20	ChiSquared 22	22	581	406	34	4094	335	97
21	ChiSquared 15	15	3593	251	26	2325	489	66
22	ChiSquared 16	16	21	250	25	45	56	65
23	ChiSquared 41	41	303	139	50	3739	329	70
24	ChiSquared 62	62	2882	404	120	197	2038	259
25	ReliefF 429	10141	12902	10141	10141	6549	429	10141
26	ReliefF 52	71	1801	438	142	11874	52	288

**FIGURE 2:**
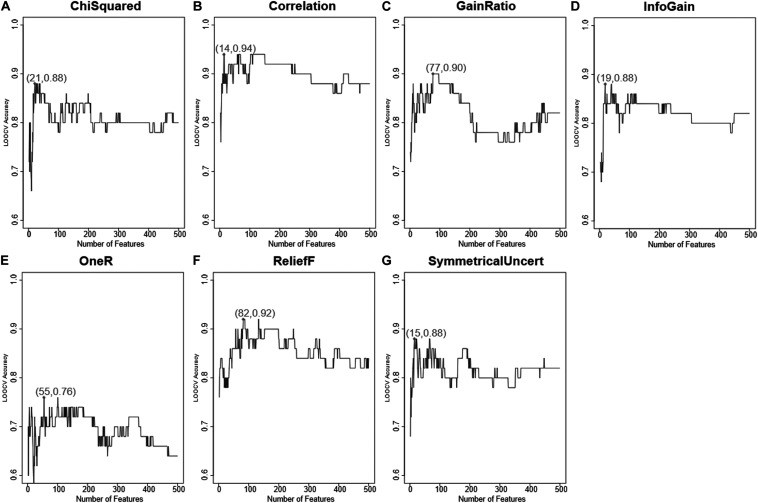
The IFS curves of seven other feature selection methods from Weka. **(A)** The IFS curve of ChiSquaredAttributeEval; **(B)** The IFS curve of CorrelationAttributeEval; **(C)** The IFS curve of GainRatioAttributeEval; **(D)** The IFS curve of InfoGainAttributeEval; **(E)** The IFS curve of OneRAttributeEval; **(F)** The IFS curve of ReliefFAttributeEval; **(G)** The IFS curve of SymmetricalUncertAttributeEval. The IFS curves of seven other feature selection methods from Weka were plotted. Their peak accuracies were 0.88, 0.94, 0.90, 0.88, 0.76, 0.92, and 0.88, all smaller than the highest accuracy of Monte-Carlo feature selection, which was 0.96.

Similarly, for the seven methods, the top 500 ranked genes were further evaluated with IFS and their accuracies were used to represent how different they were between two groups of samples. The IFS results of the seven feature selection methods in Weka was shown in Figure 2. It can be seen that the peak LOOCV SVM accuracies of ChiSquared, Correlation, GainRatio, InfoGain, OneR, ReliefF, and SymmetricalUncert were 0.88, 0.94, 0.90, 0.88, 0.76, 0.92, and 0.88, respectively. They were all smaller than the highest accuracy of Monte-Carlo feature selection, which was 0.96.

We compared the best Monte-Carlo genes with the best genes selected by the other seven methods in Weka using R package SuperExactTest^1^ (Wang et al., 2015). The number of overlapped genes between Monte-Carlo and SymmetricalUncert, ReliefF, OneR, InfoGain, GainRatio, Correlation, and ChiSquared were 10, 10, 4, 11, 13, 5, and 12, respectively. The enrichment *p* values between Monte-Carlo and SymmetricalUncert, ReliefF, OneR, InfoGain, GainRatio, Correlation, and ChiSquared were 4.88E-31, 3.41E-22, 1.78E-08, 3.85E-33, 7.46E-31, 4.57E-14, and 4.40E-36, respectively. The Monte-Carlo selected genes were most like the ChiSquared selected genes and most unlike the OneR selected genes.

### The Biological Functions of Key Genes for Perineural Invasion

The probes of Agilent-052909 CBC_lncRNAmRNA_V3 microarray were poorly annotated. We mapped the probe sequence onto the human genome using blastn^2^ with default parameters against Genome (GRCh38.p12 reference, Annotation Release 109) and identified the best match genes for these probes.

The biological functions of the 15 genes from tumor tissues, the 11 genes from adjacent tissues, and the combined 26 genes were analyzed using GATHER^3^. The enrichment results were shown in Table 3. For tumor signature genes, they were significantly enriched onto GO:0016043: cell organization and biogenesis and GO:0006996: organelle organization and biogenesis with a *p* value of 0.0004 and 0.006, respectively. For adjacent genes, TNFRSF14 was involved in hsa04060: Cytokine-cytokine receptor interaction. DOCK9, NPHP1, and SOCS4 from tumors and CREB5 and XPO1 from adjacent tissues were all targets of transcription factor NF-κB.

**TABLE 3:** The enriched functions of the 15 genes from tumor tissues, the 11 genes from adjacent tissues, and the combined 26 genes using GATHER.

		Function	# Genes	Genes	*p* value	Bayes factor
The 15 genes from tumor	Gene Ontology	GO:0016043: cell organization and biogenesis	3	HIST1H4G NPHP1 SOCS4	0.0004	4
		GO:0006996: organelle organization and biogenesis	2	HIST1H4G NPHP1	0.006	2
		V$HEN1_02: HEN1	2	NPHP1 SOCS4	0.0003	4
	TRANSFAC	V$NFKB_Q6: NF-kappaB	3	DOCK9 NPHP1 SOCS4	0.002	3
		V$HNF1_Q6	2	NPHP1 SOCS4	0.004	2
The 11 genes from adjacent	KEGG Pathway	hsa04060: Cytokine-cytokine receptor interaction	1	TNFRSF14	0.002	2
	TRANSFAC	V$USF_01: upstream stimulating factor	4	ATF3 CREB5 TNFRSF14 XPO1	<0.0001	7
		V$MYC_Q2	4	ATF3 CREB5 TNFRSF14 XPO1	0.0002	5
		V$NMYC_01: N-Myc	4	ATF3 CREB5 TNFRSF14 XPO1	0.0004	4
		V$MYCMAX_02: c-Myc:Max heterodimer	4	ATF3 CREB5 TNFRSF14 XPO1	0.001	3
		V$AML_Q6	3	ATF3 CREB5 TNFRSF14	0.002	2
		V$SRF_Q6: serum response factor	2	ATF3 CREB5	0.003	2
Combined 26 genes	TRANSFAC	V$NFKB_Q6: NF-kappaB	5	CREB5 DOCK9 NPHP1 SOCS4 XPO1	0.0008	3

Bockman DE et al. found that a large number of molecules, such as LIF (Bressy et al., 2018), CCL2–CCR2 (Jessen and Mirsky, 2016), and NCAM (Deborde et al., 2016), were involved in PNI by studying the paracrine mechanism of signal transduction between nerves and cancer cells (Wang et al., 2014). For instance, cellular adhesion molecules LICAM mediates the homologous interaction between the tumor and nerves and increases PNI to promote the development of cancer (Ben et al., 2014; Lund et al., 2015). According to Giulia Gasparini et al., nerve growth factor (NGF) may be involved in the migration of glial cells in PNI. The results suggested that high levels of NGF and its affinity receptor TrKA were associated with the frequency of occurrence and severity of PNI, as well as decreased survival time and increased pain in patients with PDAC (Barbacid, 1995; Demir et al., 2010; Wang et al., 2014; Gasparini et al., 2019). The importance of GDNF-RET signal transduction in PDAC nerve invasion has been emphasized in many studies (Gil et al., 2010; Demir et al., 2012). Demir et al. have shown that in PDAC, soluble GFRα1 released by nerves can promote the binding of neural GDNF and RET in pancreatic adenocarcinoma, thus enhancing PNI (He et al., 2014; Mulligan, 2018). The synthesis, secretion, and transport of these cytokines are carried out by organelle organization, such as ribosomes and endoplasmic reticulum (Alrawashdeh et al., 2019). This evidence supports our findings that there is a close relationship between GO:0006996 (organelle organization), hsa04060 (Cytokine-cytokine receptor interaction), and PDAC PNI. In addition, some studies have shown that the activation of the NF- κB signaling pathway affects a wide range of biological processes, including immunity, inflammation, stress response, B cell development, and lymphoid organogenesis (Yu et al., 2017; Balaji et al., 2018), while PNI in PDAC is associated with lymph node metastasis (Chatterjee et al., 2012).

We investigated their clinical relevance with the survival of 117 pancreatic ductal adenocarcinoma patients from Kaplan Meier-plotter^4^ (Nagy et al., 2018). 17 genes were included in the database. 11 of them (NPHP1, WBP2NL, EXD3, G2E3, DOCK9, CT47A12, TMEM250, PLCB1, XPO1, HIST1H4G, and SLC35E2B) were significant with a p value smaller than 0.05 and one (ATF3) was marginally significant with a *p* value of 0.074. The Kaplan Meier plot of these 12 survival-associated genes were shown in Supplementary Figure 1.

**FIGURE 3:**
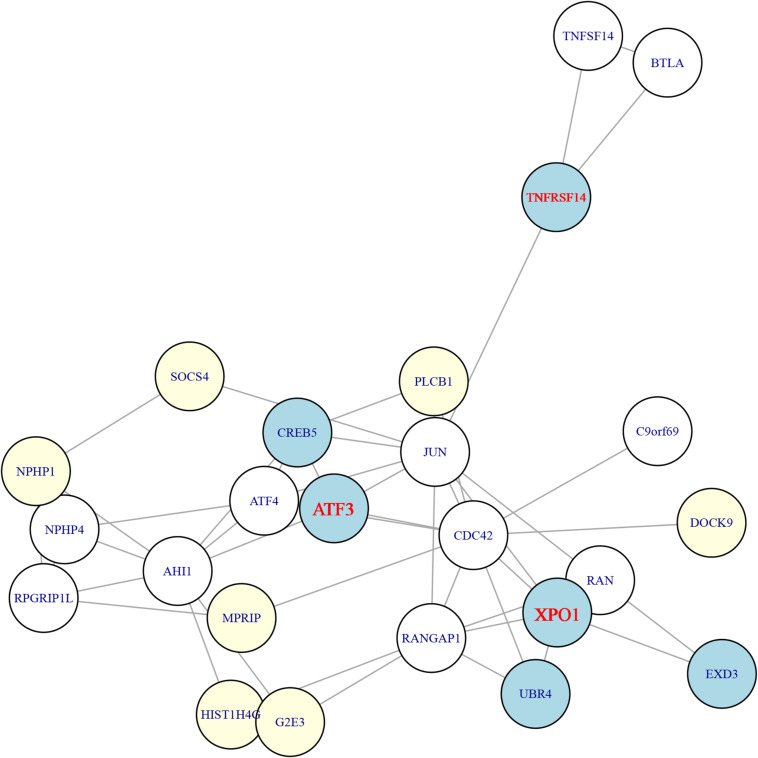
The STRING network of the 26 genes. The 26 genes were mapped onto the STRING network (https://string-db.org/). The light-yellow nodes were genes from adjacent tissues while the light-blue nodes were genes from tumor tissues. The genes from tumors can be grouped into three clusters and the hub genes of these three clusters were ATF3, XPO1, and TNFRSF14, which were highlighted in red.

To select the most possible key genes, we constructed the network of the 26 genes using STRING database^5^ version 11.0 (Szklarczyk et al., 2018). The network of the identified genes was shown in Figure 3. It can be seen that six genes from tumors were mapped onto the network and they can be grouped into three categories: (1) the XPO1, UBR4, EXD3 cluster in which XPO1 was the hub gene with degree of six (RAN, RANGAP1, CDC42, JUN, UBR4, EXD3); (2) the ATF3, CREB5 cluster in which ATF3 was the hub gene with degree of five (JUN, ATF4, CDC42, AHI1, CREB5); and (3) the TNFRSF14 cluster in which the degree of TNFRSF14 was three (JUN, BTLA, TNFSF14). Therefore, the three genes (XPO1, ATF3, and TNFRSF14) from tumors were the hubs.

Probe A_21_P0010506, ranked 5th in Table 1, was mapped onto TNFRSF14. TNFRSF14, also known as HVEM, encodes a member of the TNF receptor superfamily that activates either proinflammatory or inhibitory signaling pathways (Pasero et al., 2012). Recent reports indicate that HVEM and its ligands may also be involved in tumor progression and resistance to immune response (Derre et al., 2010). The tumor microenvironment of pancreatic cancer is rich in the expression of immune-inhibitory molecules, such as PDL1, galectin-9, HVEM, or HLA-G (Sideras et al., 2014). High tumor expression of these molecules has been identified to be associated with improved cancer-specific survival (Sideras et al., 2017). The combination of such immune biomarkers could be a powerful prognostic tool for pancreatic cancer patients, as well as targets for future immunotherapy.

Probe A_23_P40078, ranked 20th in Table 1, was mapped onto XPO1. Exportin 1 (XPO1), also called chromosome maintenance region 1 (CRM1), is known as a medium of nucleo-cytoplasmic shuttling of mature microRNAs (Muqbil et al., 2013). Recent studies show that up- or down-regulation of specific miRNAs and their target genes are directly involved in the progression and prognosis of human cancers like PDAC (Calin and Croce, 2006; Zhang et al., 2007). Azmi et al. suggested that XPO1 inhibition can down-regulate the expression of miR-145 target pathways via up-regulating the expression of tumor suppressive miR-145, therefore leading to the inhibition of migration and proliferation of PDAC (Azmi et al., 2017). High expression of XPO1, a common feature of PDAC and other cancers, results in functional inactivation of tumor suppressor proteins (TSPs) via constant nuclear protein export (Turner and Sullivan, 2008; Huang et al., 2009; Gao et al., 2014). Gao et al. developed some newly specific inhibitors of nuclear export targeting XPO1, which have been proven to inhibit the proliferation of pancreatic cancer cells and tumor invasion effectively (Gao et al., 2014). Therefore, blocking nuclear export could become an attractive therapeutic strategy for the treatment of PDAC (Mao and Yang, 2013).

Probe A_24_P33895, ranked 24th in Table 1, was mapped onto ATF3. Activating transcription factor 3 (ATF3) is involved in the complex process of cellular stress response (Hackl et al., 2010) and is a key mediator of the PERK/ATF4 pathway (Jiang et al., 2004). Several studies have identified rapid increases of ATF3 expression during pancreatic insult (Allen-Jennings et al., 2001; Hartman et al., 2004; Jiang et al., 2004), but it is still unclear how the increase affects the response of the pancreas to injury. Fazio et al. demonstrated that ATF3 reduces the severity of pancreatic injury as a key transcriptional regulator of pancreatic acinar cells (Fazio et al., 2017). However, long-term activation of ATF3 may increase the sensitivity to factors that promote PDAC.

## Conclusion

Pancreatic cancer is a common cancer and pancreatic ductal adenocarcinoma (PDAC) is the most aggressive subtype, with a 5-year survival rate of only 3–5%. Perineural invasion (PNI) is associated with the poor prognosis of PDAC. Adjacent tissues are normal tissues that grow around tumors. There are often some capillaries and immune cells in the adjacent tissues due to the influences of tumor invasion. Adjacent and tumor tissues constitute the tumor microenvironment (Degos et al., 2019; Harmon et al., 2019). Many studies have been conducted on the adjacent tissues of patients, and the results suggest that the expression of corresponding genes in adjacent tissues can be used to predict the prognosis of patients (Lee et al., 2019). To explore the mechanism of PNI, by analyzing the gene expression profiles of tumors and adjacent tissues from 28 pancreatic ductal adenocarcinoma patients with perineural invasion and 22 pancreatic ductal adenocarcinoma patients without perineural invasion, we identified 26 key features, within which 15 features were from tumor tissues while 11 features were from adjacent tissues. These results merit further validation in large cohort studies.

## Data Availability Statement

Publicly available datasets were analyzed in this study. This data can be found here: the NCBI Gene Expression Omnibus (GSE102238).

## Author Contributions

J-HZ, Q-LY, and J-WW contributed to the study design. YC conducted the literature search. Q-HY, Z-JW, and TH acquired the data. J-HZ and TH wrote the article. Q-LY and J-WW performed the data analysis. J-HZ, TH, and Q-LY revised the article and gave the final approval of the version to be submitted. All authors have read and agreed to the published version of the manuscript.

## Conflict of Interest

The authors declare that the research was conducted in the absence of any commercial or financial relationships that could be construed as a potential conflict of interest.

## Footnotes

1 https://CRAN.R-project.org/package=SuperExactTest

2 https://blast.ncbi.nlm.nih.gov

3 https://changlab.uth.tmc.edu/gather/

4 https://kmplot.com/analysis/

5 https://string-db.org/

## Supplementary Material

Supplementary Figure 1 The Kaplan Meier plot of these 12 survival-associated genes. **(A)** The Kaplan Meier plot of NPHP1; **(B)** The Kaplan Meier plot of WBP2NL; **(C)** The Kaplan Meier plot of EXD3; **(D)** The Kaplan Meier plot of G2E3; **(E)** The Kaplan Meier plot of DOCK9; **(F)** The Kaplan Meier plot of CT47A12; **(G)** The Kaplan Meier plot of TMEM250; **(H)** The Kaplan Meier plot of PLCB1; **(I)** The Kaplan Meier plot of XPO1; **(J)** The Kaplan Meier plot of HIST1H4G; **(K)** The Kaplan Meier plot of SLC35E2B; **(L)** The Kaplan Meier plot of ATF3. The clinical relevance of these genes with survival were evaluated using the 117 pancreatic ductal adenocarcinoma patients from Kaplan Meier-plotter (https://kmplot.com/analysis/).

## Abbreviations

PDAC: pancreatic ductal adenocarcinoma
PNI: perineural invasion
IFS: incremental feature selection
LOOCV: leave-one-out cross validation
GEO: Gene Expression Omnibus
TSPs: tumor suppressor proteins
ATF3: activating transcription factor 3.

## References

[B1] Allen-Jennings A. E. Hartman M. G. Kociba G. J. Hai T. (2001). The roles of ATF3 in glucose homeostasis. *A transgenic mouse model with liver dysfunction and defects in endocrine pancreas*. *J. Biol. Chem.* 276 29507–29514. 10.1074/jbc.M100986200 11371557

[B2] Alrawashdeh W. Jones R. Dumartin L. Radon T. P. Cutillas P. R. Feakins R. M. (2019). Perineural invasion in pancreatic cancer: proteomic analysis and in vitro modelling. *Mol. Oncol.* 13 1075–1091. 10.1002/1878-0261.12463 30690892 PMC6487729

[B3] Azmi A. S. Li Y. Muqbil I. Aboukameel A. Senapedis W. Baloglu E. (2017). Exportin 1 (XPO1) inhibition leads to restoration of tumor suppressor miR-145 and consequent suppression of pancreatic cancer cell proliferation and migration. *Oncotarget* 8 82144–82155. 10.18632/oncotarget.19285 29137251 PMC5669877

[B4] Balaji S. Ahmed M. Lorence E. Yan F. Nomie K. Wang M. (2018). NF-kappaB signaling and its relevance to the treatment of mantle cell lymphoma. *J. Hematol. Oncol.* 11:83 10.1186/s13045-018-0621625 PMC6002979 29907126

[B5] Bapat A. A. Hostetter G. Von Hoff D. D. Han H. (2011). Perineural invasion and associated pain in pancreatic cancer. *Nat. Rev. Cancer* 11 695–707. 10.1038/nrc3131 21941281

[B6] Barbacid M. (1995). Structural and functional properties of the TRK family of neurotrophin receptors. *Ann. N Y Acad. Sci.* 766 442–458. 10.1111/j.1749-6632.1995.tb26693.x 7486690

[B7] Ben Q. An W. Fei J. Xu M. Li G. Li Z. (2014). Downregulation of L1CAM inhibits proliferation, invasion and arrests cell cycle progression in pancreatic cancer cells in vitro. *Exp. Ther. Med.* 7 785–790. 10.3892/etm.2014.1519 24660028 PMC3961134

[B8] Bressy C. Lac S. Nigri J. Leca J. Roques J. Lavaut M. N. (2018). LIF Drives Neural Remodeling in Pancreatic Cancer and Offers a New Candidate Biomarker. *Cancer Res* 78 909–921. 10.1158/0008-5472.Can-152790 29269518

[B9] Cai S. Hong T. S. Goldberg S. I. Fernandez-del Castillo C. Thayer S. P. Ferrone C. R. (2013). Updated long-term outcomes and prognostic factors for patients with unresectable locally advanced pancreatic cancer treated with intraoperative radiotherapy at the Massachusetts General Hospital, 1978 to 2010. *Cancer* 119 4196–4204. 10.1002/cncr.28329 24006012 PMC4403862

[B10] Calabretta S. Bielli P. Passacantilli I. Pilozzi E. Fendrich V. Capurso G. (2016). Modulation of PKM alternative splicing by PTBP1 promotes gemcitabine resistance in pancreatic cancer cells. *Oncogene* 35 2031–2039. 10.1038/onc.2015.270 26234680 PMC4650269

[B11] Calin G. A. Croce C. M. (2006). MicroRNA signatures in human cancers. *Nat. Rev. Cancer* 6 857–866. 10.1038/nrc1997 17060945

[B12] Campbell P. M. Groehler A. L. Lee K. M. Ouellette M. M. Khazak V. Der C. J. (2007). K-Ras promotes growth transformation and invasion of immortalized human pancreatic cells by Raf and phosphatidylinositol 3-kinase signaling. *Cancer Res.* 67 2098–2106. 10.1158/0008-5472.can-063752 17332339

[B13] Casbas-Hernandez P. Sun X. Roman-Perez E. D’Arcy M. Sandhu R. Hishida A. (2015). Tumor intrinsic subtype is reflected in cancer-adjacent tissue. *Cancer Epidemiol. Biomark. Prev.* 24 406–414. 10.1158/1055-9965.Epi-140934 PMC4437571 25465802

[B14] Ceyhan G. O. Demir I. E. Altintas B. Rauch U. Thiel G. Muller M. W. (2008). Neural invasion in pancreatic cancer: a mutual tropism between neurons and cancer cells. *Biochem. Biophys. Res. Commun.* 374 442–447. 10.1016/j.bbrc.2008.07.035 18640096

[B15] Chatterjee D. Katz M. H. Rashid A. Wang H. Iuga A. C. Varadhachary G. R. (2012). Perineural and intraneural invasion in posttherapy pancreaticoduodenectomy specimens predicts poor prognosis in patients with pancreatic ductal adenocarcinoma. *Am. J. Surg. Pathol.* 36 409–417. 10.1097/PAS.0b013e31824104c5 22301497 PMC3288807

[B16] Chen L. Li J. Zhang Y. H. Feng K. Wang S. Zhang Y. (2018a). Identification of gene expression signatures across different types of neural stem cells with the Monte-Carlo feature selection method. *J. Cell Biochem.* 119 3394–3403. 10.1002/jcb.26507 29130544

[B17] Chen L. Pan X. Hu X. Zhang Y. H. Wang S. Huang T. (2018b). Gene expression differences among different MSI statuses in colorectal cancer. *Int. J. Cancer* 143(7), 1731–1740. 10.1002/ijc.31554 29696646

[B18] Chen L. Wang S. Zhang Y. H. Wei L. Xu X. Huang T. (2018c). Prediction of nitrated tyrosine residues in protein sequences by extreme learning machine and feature selection methods. *Comb. Chem. High Throug. Screen* 21(6), 393–402. 10.2174/1386207321666180531091619 29848272

[B19] Chen L. Wang S. Zhang Y. H. Li J. Xing Z. H. Yang J. (2017a). Identify key sequence features to improve CRISPR sgRNA efficacy. *IEEE Access.* 99 1–1. 10.1109/ACCESS.2017.2775703

[B20] Chen L. Zhang Y.-H. Huang G. Pan X. Wang S. Huang T. (2017b). Discriminating cirRNAs from other lncRNAs using a hierarchical extreme learning machine (H-ELM) algorithm with feature selection. *Mole. Gen. Genom.* 293(1), 137–149 10.1007/s00438-017-1372-7 28913654

[B21] Chen L. Zhang Y.-H. Lu G. Huang T. Cai Y.-D. (2017c). Analysis of cancer-related lncRNAs using gene ontology and KEGG pathways. *Artif. Intell. Med.* 76 27–36. 10.1016/j.artmed.2017.02.001 28363286

[B22] Chen L. Zhang Y.-H. Wang S. Zhang Y. Huang T. Cai Y.-D. (2017d). Prediction and analysis of essential genes using the enrichments of gene ontology and KEGG pathways. *PLoS One* 12:e0184129. 10.1371/journal.pone.0184129 28873455 PMC5584762

[B23] Cid-Arregui A. Juarez V. (2015). Perspectives in the treatment of pancreatic adenocarcinoma. *World J. Gastr.* 21 9297–9316. 10.3748/wjg.v21.i31.9297 26309356 PMC4541382

[B24] Dai H. Li R. Wheeler T. Ozen M. Ittmann M. Anderson M. (2007). Enhanced survival in perineural invasion of pancreatic cancer: an in vitro approach. *Hum. Pathol.* 38 299–307. 10.1016/j.humpath.2006.08.002 17097719

[B25] De Oliveira T. Abiatari I. Raulefs S. Sauliunaite D. Erkan M. Kong B. (2012). Syndecan-2 promotes perineural invasion and cooperates with K-ras to induce an invasive pancreatic cancer cell phenotype. *Mol. Cancer* 11:19 10.1186/1476-4598-1119 PMC3350462 22471946

[B26] Deborde S. Omelchenko T. Lyubchik A. Zhou Y. He S. McNamara W. F. (2016). Schwann cells induce cancer cell dispersion and invasion. *J. Clin. Invest.* 126 1538–1554. 10.1172/jci82658 26999607 PMC4811155

[B27] Degos C. Heinemann M. Barrou J. Boucherit N. Lambaudie E. Savina A. (2019). Endometrial Tumor Microenvironment Alters Human NK Cell Recruitment, and Resident NK Cell Phenotype and Function. *Front. Immunol.* 10:877. 10.3389/fimmu.2019.00877 31105699 PMC6498896

[B28] Demir I. E. Ceyhan G. O. Liebl F. D’Haese J. G. Maak M. Friess H. (2010). Neural invasion in pancreatic cancer: the past, present and future. *Cancers* 2 1513–1527. 10.3390/cancers2031513 24281170 PMC3837319

[B29] Demir I. E. Friess H. Ceyhan G. O. (2012). Nerve-cancer interactions in the stromal biology of pancreatic cancer. *Front. Physiol.* 3:97. 10.3389/fphys.2012.00097 22529816 PMC3327893

[B30] Derre L. Rivals J. P. Jandus C. Pastor S. Rimoldi D. Romero P. (2010). BTLA mediates inhibition of human tumor-specific CD8+ T cells that can be partially reversed by vaccination. *J. Clin. Invest.* 120 157–167. 10.1172/jci40070 20038811 PMC2799219

[B31] Draminski M. Rada-Iglesias A. Enroth S. Wadelius C. Koronacki J. Komorowski J. (2008). Monte Carlo feature selection for supervised classification. *Bioinformatics* 24 110–117. 10.1093/bioinformatics/btm486 18048398

[B32] Esposito I. Kleeff J. Bergmann F. Reiser C. Herpel E. Friess H. (2008). Most pancreatic cancer resections are R1 resections. *Ann. Surg. Oncol.* 15 1651–1660. 10.1245/s10434-008-98399838 18351300

[B33] Fazio E. N. Young C. C. Toma J. Levy M. Berger K. R. Johnson C. L. (2017). Activating transcription factor 3 promotes loss of the acinar cell phenotype in response to cerulein-induced pancreatitis in mice. *Mol. Biol. Cell.* 28 2347–2359. 10.1091/mbc.E17-040254 28701342 PMC5576899

[B34] Ferrone C. R. Marchegiani G. Hong T. S. Ryan D. P. Deshpande V. McDonnell E. I. (2015). Radiological and surgical implications of neoadjuvant treatment with FOLFIRINOX for locally advanced and borderline resectable pancreatic cancer. *Ann. Surg.* 261 12–17. 10.1097/sla.0000000000000867 25599322 PMC4349683

[B35] Frank E. Hall M. A. Witten I. H. (2016). *The WEKA Workbench. Online Appendix for “Data Mining: Practical Machine Learning Tools and Techniques.* New Delhi: Elsevier.

[B36] Gao J. Azmi A. S. Aboukameel A. Kauffman M. Shacham S. Abou-Samra A. B. (2014). Nuclear retention of Fbw7 by specific inhibitors of nuclear export leads to Notch1 degradation in pancreatic cancer. *Oncotarget* 5 3444–3454. 10.18632/oncotarget.1813 24899509 PMC4116494

[B37] Gasparini G. Pellegatta M. Crippa S. Lena M. S. Belfiori G. Doglioni C. (2019). Nerves and Pancreatic Cancer: New Insights into a Dangerous Relationship. *Cancers* 11:893. 10.3390/cancers11070893 31248001 PMC6678884

[B38] Gil Z. Cavel O. Kelly K. Brader P. Rein A. Gao S. P. (2010). Paracrine regulation of pancreatic cancer cell invasion by peripheral nerves. *J. Natl. Cancer Inst.* 102 107–118. 10.1093/jnci/djp456 20068194 PMC2911041

[B39] Giulietti M. Occhipinti G. Principato G. Piva F. (2016). Weighted gene co-expression network analysis reveals key genes involved in pancreatic ductal adenocarcinoma development. *Cell Oncol.* 39 379–388. 10.1007/s13402-016-0283287 PMC13001876 27240826

[B40] Gohrig A. Detjen K. M. Hilfenhaus G. Korner J. L. Welzel M. Arsenic R. (2014). Axon guidance factor SLIT2 inhibits neural invasion and metastasis in pancreatic cancer. *Cancer Res.* 74 1529–1540. 10.1158/0008-5472.can-131012 24448236

[B41] Hackl C. Lang S. A. Moser C. Mori A. Fichtner-Feigl S. Hellerbrand C. (2010). Activating transcription factor-3 (ATF3) functions as a tumor suppressor in colon cancer and is up-regulated upon heat-shock protein 90 (Hsp90) inhibition. *BMC Cancer* 10:668 10.1186/1471-2407-10668 PMC3003660 21129190

[B42] Harmon C. Robinson M. W. Hand F. Almuaili D. Mentor K. Houlihan D. D. (2019). Lactate-Mediated Acidification of Tumor Microenvironment Induces Apoptosis of Liver-Resident NK Cells in Colorectal Liver Metastasis. *Cancer Immunol. Res.* 7 335–346. 10.1158/2326-6066.Cir-180481 30563827

[B43] Hartman M. G. Lu D. Kim M. L. Kociba G. J. Shukri T. Buteau J. (2004). Role for activating transcription factor 3 in stress-induced beta-cell apoptosis. *Mol. Cell Biol.* 24 5721–5732. 10.1128/mcb.24.13.5721-5732.2004 15199129 PMC480886

[B44] He S. Chen C. H. Chernichenko N. He S. Bakst R. L. Barajas F. (2014). GFRalpha1 released by nerves enhances cancer cell perineural invasion through GDNF-RET signaling. *Proc. Natl. Acad. Sci. U S A* 111 E2008–E2017. 10.1073/pnas.1402944111 24778213 PMC4024863

[B45] Hirai I. Kimura W. Ozawa K. Kudo S. Suto K. Kuzu H. (2002). Perineural invasion in pancreatic cancer. *Pancreas* 24 15–25. 11741178 10.1097/00006676-200201000-00003

[B46] Huang W. Y. Yue L. Qiu W. S. Wang L. W. Zhou X. H. Sun Y. J. (2009). Prognostic value of CRM1 in pancreas cancer. *Clin. Invest. Med.* 32:E315. 20003838

[B47] Huang Y. K. Liu H. Wang X. Z. Zhu S. (2014). Annexin A2 and CD105 expression in pancreatic ductal adenocarcinoma is associated with tumor recurrence and prognosis. *Asian Pac. J. Cancer Prev.* 15 9921–9926. 10.7314/apjcp.2014.15.22.9921 25520129

[B48] Jessen K. R. Mirsky R. (2016). The repair Schwann cell and its function in regenerating nerves. *J. Physiol.* 594 3521–3531. 10.1113/jp270874 26864683 PMC4929314

[B49] Jiang H. Y. Wek S. A. McGrath B. C. Lu D. Hai T. Harding H. P. (2004). Activating transcription factor 3 is integral to the eukaryotic initiation factor 2 kinase stress response. *Mol. Cell Biol.* 24 1365–1377. 10.1128/mcb.24.3.1365-1377.2004 14729979 PMC321431

[B50] Kleger A. Perkhofer L. Seufferlein T. (2014). Smarter drugs emerging in pancreatic cancer therapy. *Ann. Oncol.* 25 1260–1270. 10.1093/annonc/mdu013 24631947

[B51] Lee J. W. Kim S. Y. Lee H. J. Han S. W. Lee J. E. Lee S. M. (2019). Prognostic Significance of CT-Attenuation of Tumor-Adjacent Breast Adipose Tissue in Breast Cancer Patients with Surgical Resection. *Cancers* 11:11335. 10.3390/cancers11081135 31398863 PMC6721593

[B52] Li J. Huang T. (2017). Predicting and analyzing early wake-up associated gene expressions by integrating GWAS and eQTL studies. *Biochim. Biophy. Acta Mole. Basis Dis.* 1864 2241–2246. 10.1016/j.bbadis.2017.10.036 29109033

[B53] Li Q. Xia S. Yin Y. Guo Y. Chen F. Jin P. (2018). miR-5591-5p regulates the effect of ADSCs in repairing diabetic wound via targeting AGEs/AGER/JNK signaling axis. *Cell Death Dis.* 9:566 10.1038/s41419-018-0615619 PMC5948214 29752466

[B54] Liu L. Chen L. Zhang Y. H. Wei L. Cheng S. Kong X. (2017). Analysis and prediction of drug-drug interaction by minimum redundancy maximum relevance and incremental feature selection. *J. Biomole. Struct. Dynam.* 35 312–329. 10.1080/07391102.2016.1138142 26750516

[B55] Lund K. Dembinski J. L. Solberg N. Urbanucci A. Mills I. G. Krauss S. (2015). Slug-dependent upregulation of L1CAM is responsible for the increased invasion potential of pancreatic cancer cells following long-term 5-FU treatment. *PLoS One* 10:e0123684. 10.1371/journal.pone.0123684 25860483 PMC4393253

[B56] Mao L. Yang Y. (2013). Targeting the nuclear transport machinery by rational drug design. *Curr. Pharm. Des.* 19 2318–2325. 10.2174/1381612811319120018 23082981

[B57] Mossner J. (2010). What’s new in therapy of pancreatic cancer? *Dig. Dis.* 28 679–683. 10.1159/000320096 21088420

[B58] Mulligan L. M. (2018). GDNF and the RET Receptor in Cancer: New Insights and Therapeutic Potential. *Front. Physiol.* 9:1873. 10.3389/fphys.2018.01873 30666215 PMC6330338

[B59] Muqbil I. Bao B. Abou-Samra A. B. Mohammad R. M. Azmi A. S. (2013). Nuclear export mediated regulation of microRNAs: potential target for drug intervention. *Curr. Drug. Target.* 14 1094–1100. 10.2174/1389450111314100002 23834155 PMC4167361

[B60] Nagy Á Lánczky A. Menyhárt O. Gyõrffy B. (2018). Validation of miRNA prognostic power in hepatocellular carcinoma using expression data of independent datasets. *Sci. Rep.* 8:9227. 10.1038/s41598-018-27521-y 29907753 PMC6003936

[B61] Nakao A. Harada A. Nonami T. Kaneko T. Takagi H. (1996). Clinical significance of carcinoma invasion of the extrapancreatic nerve plexus in pancreatic cancer. *Pancreas* 12 357–361. 10.1097/00006676-199605000-00006 8740402

[B62] Pan X. Hu X. Zhang Y. H. Feng K. Wang S. P. Chen L. (2018). Identifying Patients with Atrioventricular Septal Defect in Down Syndrome Populations by Using Self-Normalizing Neural Networks and Feature Selection. *Genes* 9:208. 10.3390/genes9040208 29649131 PMC5924550

[B63] Pasero C. Speiser D. E. Derre L. Olive D. (2012). The HVEM network: new directions in targeting novel costimulatory/co-inhibitory molecules for cancer therapy. *Curr. Opin. Pharmacol.* 12 478–485. 10.1016/j.coph.2012.03.001 22445654

[B64] Pour P. M. Bell R. H. Batra S. K. (2003). Neural invasion in the staging of pancreatic cancer. *Pancreas* 26 322–325. 10.1097/00006676-200305000-00002 12717262

[B65] Rossi M. L. Rehman A. A. Gondi C. S. (2014). Therapeutic options for the management of pancreatic cancer. *World J. Gastroenterol.* 20 11142–11159. 10.3748/wjg.v20.i32.11142 25170201 PMC4145755

[B66] Sideras K. Biermann K. Yap K. Mancham S. Boor P. P. C. Hansen B. E. (2017). Tumor cell expression of immune inhibitory molecules and tumor-infiltrating lymphocyte count predict cancer-specific survival in pancreatic and ampullary cancer. *Int. J. Cancer* 141 572–582. 10.1002/ijc.30760 28470686

[B67] Sideras K. Braat H. Kwekkeboom J. van Eijck C. H. Peppelenbosch M. P. Sleijfer S. (2014). Role of the immune system in pancreatic cancer progression and immune modulating treatment strategies. *Cancer Treat. Rev.* 40 513–522. 10.1016/j.ctrv.2013.11.005 24315741

[B68] Szklarczyk D. Gable A. L. Lyon D. Junge A. Wyder S. Huerta-Cepas J. (2018). STRING v11: protein-protein association networks with increased coverage, supporting functional discovery in genome-wide experimental datasets. *Nucl. Acids Res.* 47:542. 10.1093/nar/gky1131 30476243 PMC6323986

[B69] Turner J. G. Sullivan D. M. (2008). CRM1-mediated nuclear export of proteins and drug resistance in cancer. *Curr. Med. Chem.* 15 2648–2655. 10.2174/092986708786242859 18991627

[B70] Wang D. Li J.-R. Zhang Y.-H. Chen L. Huang T. Cai Y.-D. (2018). Identification of Differentially Expressed Genes between Original Breast Cancer and Xenograft Using Machine Learning Algorithms. *Genes* 9:155. 10.3390/genes9030155 29534550 PMC5867876

[B71] Wang K. Demir I. E. D’Haese J. G. Tieftrunk E. Kujundzic K. Schorn S. (2014). The neurotrophic factor neurturin contributes toward an aggressive cancer cell phenotype, neuropathic pain and neuronal plasticity in pancreatic cancer. *Carcinogenesis* 35 103–113. 10.1093/carcin/bgt312 24067900

[B72] Wang M. Zhao Y. Zhang B. (2015). Efficient Test and Visualization of Multi-Set Intersections. *Sci. Rep.* 5:16923. 10.1038/srep16923 26603754 PMC4658477

[B73] Wang S. Wang D. Li J. Huang T. Cai Y.-D. (2017). Identification and analysis of the cleavage site in signal peptide by using SMOTE, Dagging, and feature selection methods. *Mole. Omics* 14(1), 64–73. 10.1039/c7mo00030h 29725682

[B74] White R. R. Hurwitz H. I. Morse M. A. Lee C. Anscher M. S. Paulson E. K. (2001). Neoadjuvant chemoradiation for localized adenocarcinoma of the pancreas. *Ann. Surg. Oncol.* 8 758–765. 11776488 10.1007/s10434-001-0758-1

[B75] Yamakawa N. Kirita T. Umeda M. Yanamoto S. Ota Y. Otsuru M. (2019). Tumor budding and adjacent tissue at the invasive front correlate with delayed neck metastasis in clinical early-stage tongue squamous cell carcinoma. *J. Surg. Oncol.* 119 370–378. 10.1002/jso.25334 30548537 PMC6590300

[B76] Yang M. W. Tao L. Y. Jiang Y. S. Yang J. Y. Huo Y. M. Liu D. J. (2020). Perineural Invasion Reprograms the Immune Microenvironment through Cholinergic Signaling in Pancreatic Ductal Adenocarcinoma. *Cancer Res.* 80 1991–2003. 10.1158/0008-5472.Can-192689 32098780

[B77] Yu L. Li L. Medeiros L. J. Young K. H. (2017). NF-kappaB signaling pathway and its potential as a target for therapy in lymphoid neoplasms. *Blood Rev.* 31 77–92. 10.1016/j.blre.2016.10.001 27773462 PMC5382109

[B78] Zhang B. Pan X. Cobb G. P. Anderson T. A. (2007). microRNAs as oncogenes and tumor suppressors. *Dev. Biol.* 302 1–12. 10.1016/j.ydbio.2006.08.028 16989803

[B79] Zhang J. Fei B. Wang Q. Song M. Yin Y. Zhang B. (2014). MicroRNA-638 inhibits cell proliferation, invasion and regulates cell cycle by targeting tetraspanin 1 in human colorectal carcinoma. *Oncotarget* 5 12083–12096. 10.18632/oncotarget.2499 25301729 PMC4322991

[B80] Zhang Y. H. Huang T. Chen L. Xu Y. Hu Y. Hu L. D. (2017). Identifying and analyzing different cancer subtypes using RNA-seq data of blood platelets. *Oncotarget* 8 87494–87511. 10.18632/oncotarget.20903 29152097 PMC5675649

[B81] Zhu Z. Friess H. diMola F. F. Zimmermann A. Graber H. U. Korc M. (1999). Nerve growth factor expression correlates with perineural invasion and pain in human pancreatic cancer. *J. Clin. Oncol.* 17 2419–2428. 10.1200/jco.1999.17.8.2419 10561305

