# Supplementary figures and images for: The Key Genes for Perineural Invasion in Pancreatic Ductal Adenocarcinoma Identified With Monte-Carlo Feature Selection Method
Source: Front Genet. 2020 Oct 15;11:554502. doi: 10.3389/fgene.2020.554502 (PMC7593847; doi:10.3389/fgene.2020.554502)

A

NPHP1

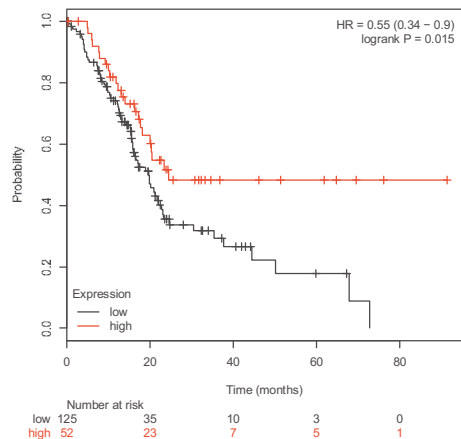

B

WBP2NL

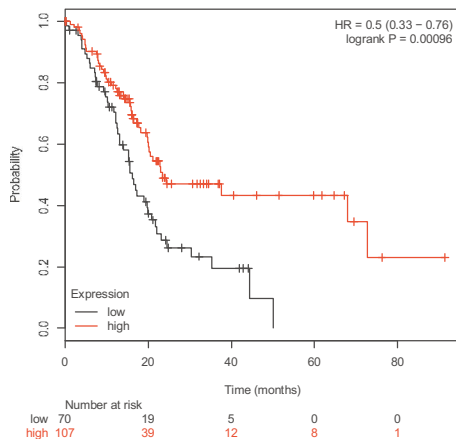

C

EXD3

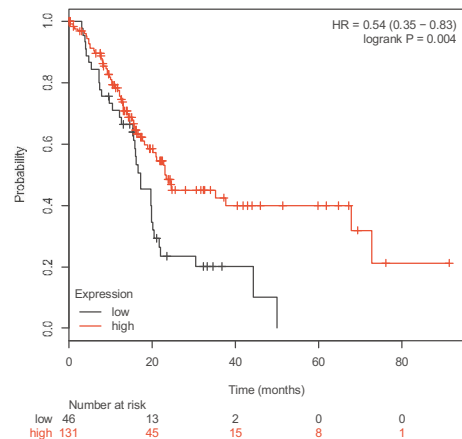

D

G2E3

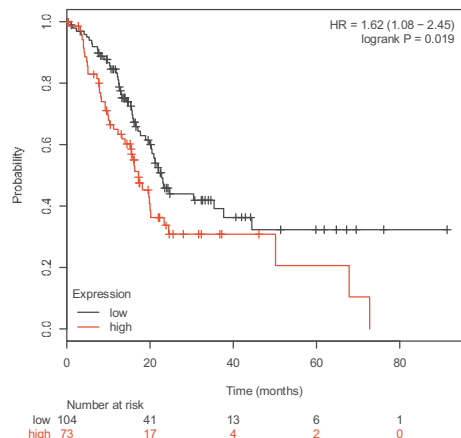

E

DOCK9

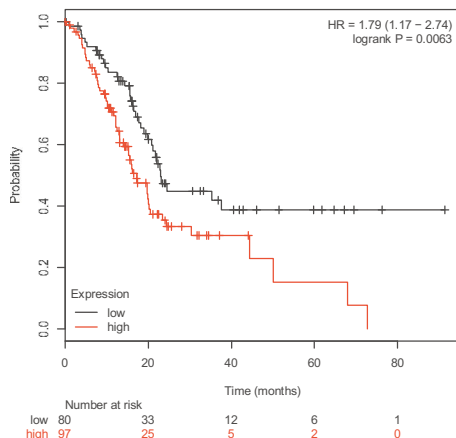

F

CT47A12

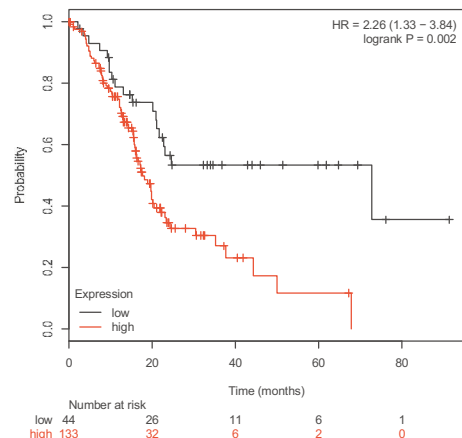

G

TMEM250

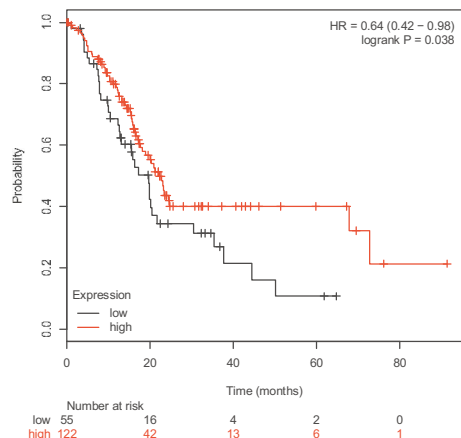

H

PLCB1

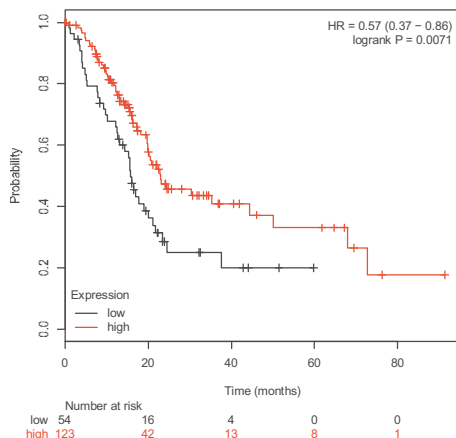

I

XPO1

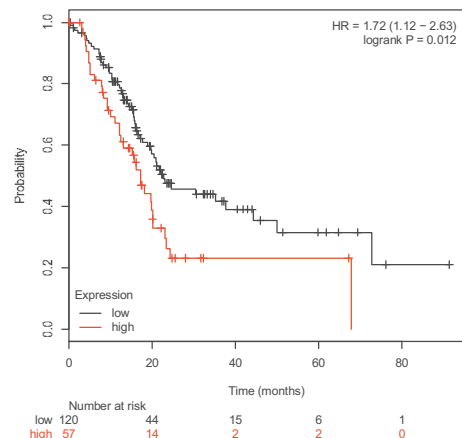

J

HIST1H4G

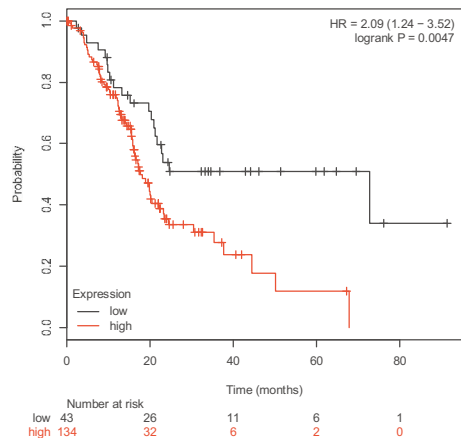

K

SLC35E2B

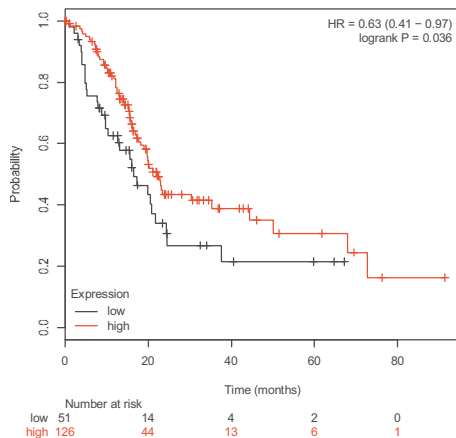

L

ATF3

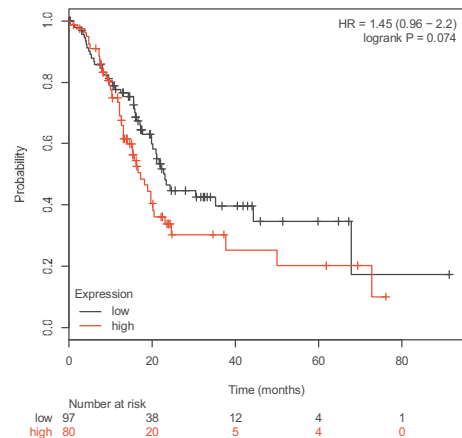

Supplement: Supplementary Figure 1 — The Kaplan Meier plot of these 12 survival-associated genes. (A) The Kaplan Meier plot of NPHP1; (B) The Kaplan Meier plot of WBP2NL; (C) The Kaplan Meier plot of EXD3; (D) The Kaplan Meier plot of G2E3; (E) The Kaplan Meier plot of DOCK9; (F) The Kaplan Meier plot of CT47A12; (G) The Kaplan Meier plot of TMEM250; (H) The Kaplan Meier plot of PLCB1; (I) The Kaplan Meier plot of XPO1; (J) The Kaplan Meier plot of HIST1H4G; (K) The Kaplan Meier plot of SLC35E2B; (L) The Kaplan Meier plot of ATF3. The clinical relevance of these genes with survival were evaluated using the 117 pancreatic ductal adenocarcinoma patients from Kaplan Meier-plotter (https://kmplot.com/analysis/). [file Image_1.pdf]
